# Different effects of hydrogen-rich water intake and hydrogen gas inhalation on gut microbiome and plasma metabolites of rats in health status

**DOI:** 10.1038/s41598-022-11091-1

**Published:** 2022-05-04

**Authors:** Fei Xie, Xue Jiang, Yang Yi, Zi-Jia Liu, Chen Ma, Jin He, Zhi-ming Xun, Meng Wang, Meng-yu Liu, Yao Mawulikplimi Adzavon, Peng-xiang Zhao, Xue-mei Ma

**Affiliations:** 1 Faculty of Environment and Life, Beijing University of Technology, No. 100, Pingleyuan, Chaoyang District, Beijing, 100124 China; 2 Beijing Molecular Hydrogen Research Center, Beijing, 100124 China

**Keywords:** Biochemistry, Molecular biology

## Abstract

The potential for preventive and therapeutic applications of H_2_ have now been confirmed in various disease. However, the effects of H_2_ on health status have not been fully elucidated. Our previous study reported changes in the body weight and 13 serum biochemical parameters during the six-month hydrogen intervention. To obtain a more comprehensive understanding of the effects of long-term hydrogen consumption, the plasma metabolome and gut microbiota were investigated in this study. Compared with the control group, 14 and 10 differential metabolites (DMs) were identified in hydrogen-rich water (HRW) and hydrogen inhalation (HI) group, respectively. Pathway enrichment analysis showed that HRW intake mainly affected starch and sucrose metabolism, and DMs in HI group were mainly enriched in arginine biosynthesis. 16S rRNA gene sequencing showed that HRW intake induced significant changes in the structure of gut microbiota, while no marked bacterial community differences was observed in HI group. HRW intake mainly induced significant increase in the abundance of *Lactobacillus*, *Ruminococcus*, *Clostridium XI*, and decrease in *Bacteroides*. HI mainly induced decreased abundances of *Blautia* and *Paraprevotella*. The metabolic function was determined by metabolic cage analysis and showed that HI decreased the voluntary intake and excretions of rats, while HRW intake did not. The results of this study provide basic data for further research on hydrogen medicine. Determination of the effects of hydrogen intervention on microbiota profiles could also shed light on identification of mechanism underlying the biological effects of molecular hydrogen.

## Introduction

Hydrogen (H_2_) is the smallest and lightest gas molecule, which has been historically considered as a biologically inert molecule. Early in 1975, Dole et al. firstly reported the possible anti-cancer effect of hyperbaric treatment of 97.5% hydrogen gas in a mouse model of skin tumor^1^. However, medical researchers did not pay considerable attention to H_2_ until Ohsawa et al. reported that inhalation of 1–4% H_2_ gas significantly attenuates cerebral ischemia–reperfusion injury in rats by selectively neutralizing hydroxyl radicals and peroxynitrite^2^. The potential for preventive and therapeutic applications of H_2_ have now been confirmed in more than 170 different human and animal-disease models, including ischemia–reperfusion (I/R) injuries^3,4^, neurodegeneration^5,6^, cardiovascular diseases^7,8^, metabolic syndrome^9,10^, inflammation^11,12^, and cancer^13,14^. Several biological mechanisms have been proposed, including selectively reducing cytotoxic oxygen radicals^2^, anti-inflammatory effects^15^, recovering mitochondrial dysfunction^16^, regulation of endoplasmic reticulum stress^17^, but none of them can fully explain the multiple biological functions of H_2_.

In mammals, the gut microbiome forms a complex ecosystem consisting of a vast number of interacting bacteria, archaea, bacteriophages, eukaryotic virus and fungi, most of which are commensal or mutualistic microorganisms^18^. In the past decade the gut microbiota has been proved to play a profound role in the training of host immunity, digesting food, regulating gut endocrine function and neurological signalling, modifying drug action and metabolism, eliminating toxins and producing numerous compounds that influence the host^19^. At present, the research on the relationship between hydrogen consumption and gut microbiome is relatively limited. Most studies showed that hydrogen-rich water (HRW) could improve intestinal structural integrity and upregulation of butyrate-producing bacteria with ameliorated clinical features of gut microbiota disturbances^20^. However, these studies have been primarily focused on the modulatory effect of HRW consumption on intestinal flora in pathological conditions, whether administration of HRW regulates gut microbiome in healthy animals remains largely unknown. In addition, as another commonly used method of hydrogen consumption, whether hydrogen inhalation could also affect gut microbiome need to be further investigated.

One of the aims of the present study was to explore the possible regulatory effects of long term of HRW intake and hydrogen inhalation on gut microbiome. Our previous study showed that long term of HRW intake or hydrogen inhalation can influence some serum biochemical parameters of normal rats, indicating the potential modulatory effect of hydrogen consumption on metabolism in health status^21^. To further investigate the effect of hydrogen consumption on metabolism, LC–MS based pseudotargeted metabolomics analysis was performed to determine the changes in the levels of plasma metabolites. The relationship between differentially expressed plasma metabolites and altered gut microbiota was also investigated.

## Results

### Effects of hydrogen intervention on body weight, voluntary intake and excretions of rats

**Figure 1:**
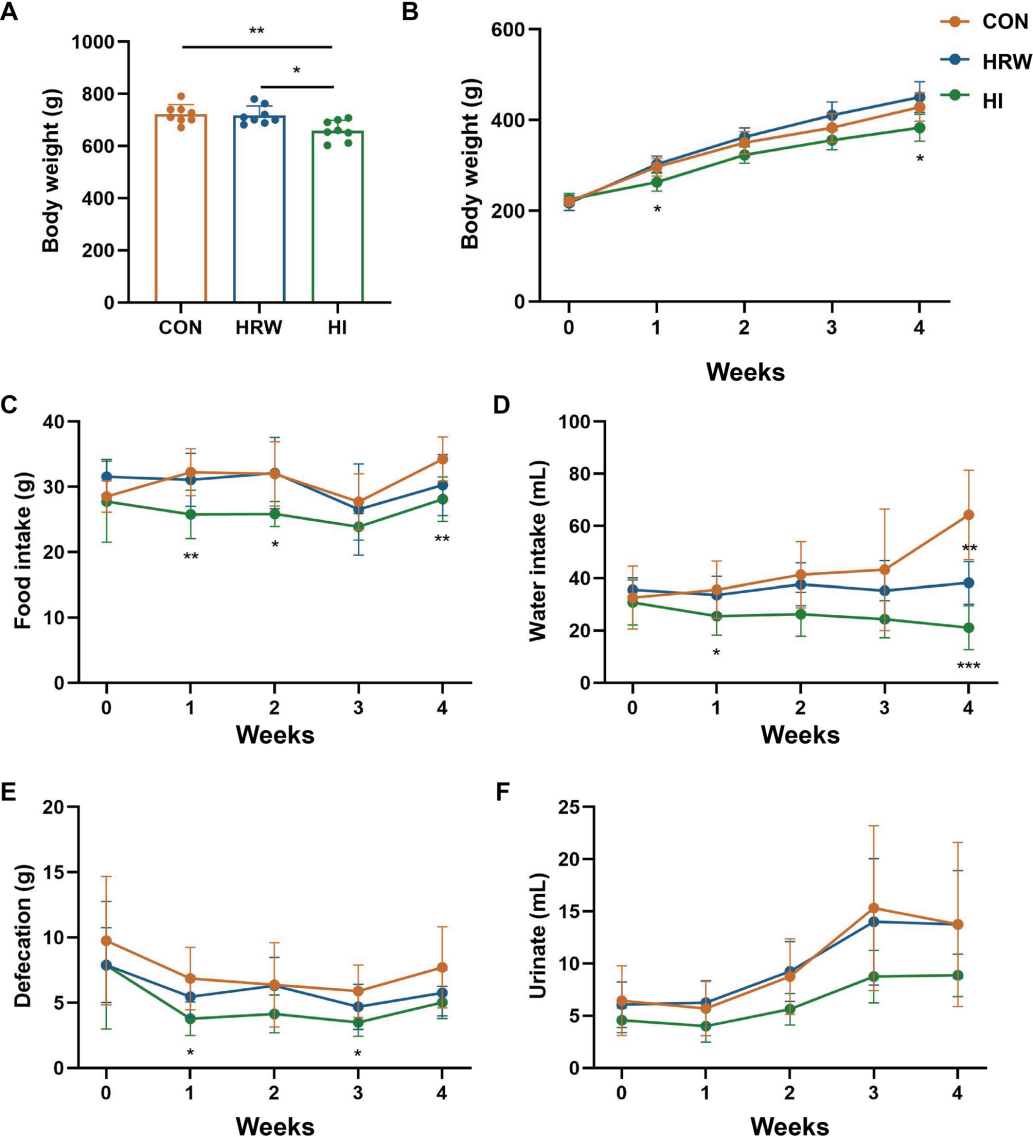
The effects of hydrogen intervention on the BW and metabolic function of rats. (**A**) BW changes at six month after hydrogen treatment; (**B**) BW changes during the first month after hydrogen treatment; (**C**–**F**) the effects of hydrogen treatment on food intake (**C**), water intake (**D**), defecation (**E**), and urinate (**F**).

As reported in our previous study^21^, after 6-month hydrogen intervention, the body weights (BW) of rats in HI group were significantly decreased (455.20 ± 31.57 g vs. 525.00 ± 17.78 g, p = 0.007) compared with the controls, while no significant changes in BW were observed in HRW group. The BW of rats were lower in HI group than in HRW group (455.20 ± 31.57 g vs. 498.80 ± 18.50 g, p = 0.012) (Fig. 1A).

To determine the effects of hydrogen intervention on metabolic function, we conducted another study using metabolic cages to measure 24-h voluntary intake and excretions of rats during the first month of hydrogen intervention. Compared with the control group, both HRW and HI had no significant effect on the BW of rats during the four weeks, however, the BW was significantly lower at the first (263.29 ± 19.98 g vs. 296.56 ± 19.69 g, p = 0.012) and the fourth week (383.21 ± 29.36 g vs. 428.81 ± 31.21 g, p = 0.024) after HI. Compared with HRW group, the BW of rats in HI group was lower with a marginal significance (p = 0.063) during the four weeks (Fig. 1B). As shown in Fig. 1C,D, compared with the control group, HI induced a significant decrease in both food intake (p < 0.0001) and water intake (p < 0.0001), while no reduction was observed in HRW group. During the four weeks, the defecation (p = 0.002, Fig. 1E) and urination (p = 0.006, Fig. 1F) was also markedly decreased in HI group, while no effect was observed in HRW group. Spearman’s correlation analysis revealed that the reduced defecation of rats in HI group was positively related to the reduction of food intake (r = 0.43, p = 0.006), and the decreased urinate was also positively related to the reduced water intake (r = 0.64, p < 0.0001).

### Effects of hydrogen intervention on plasma metabolites of rats

**Figure 2:**
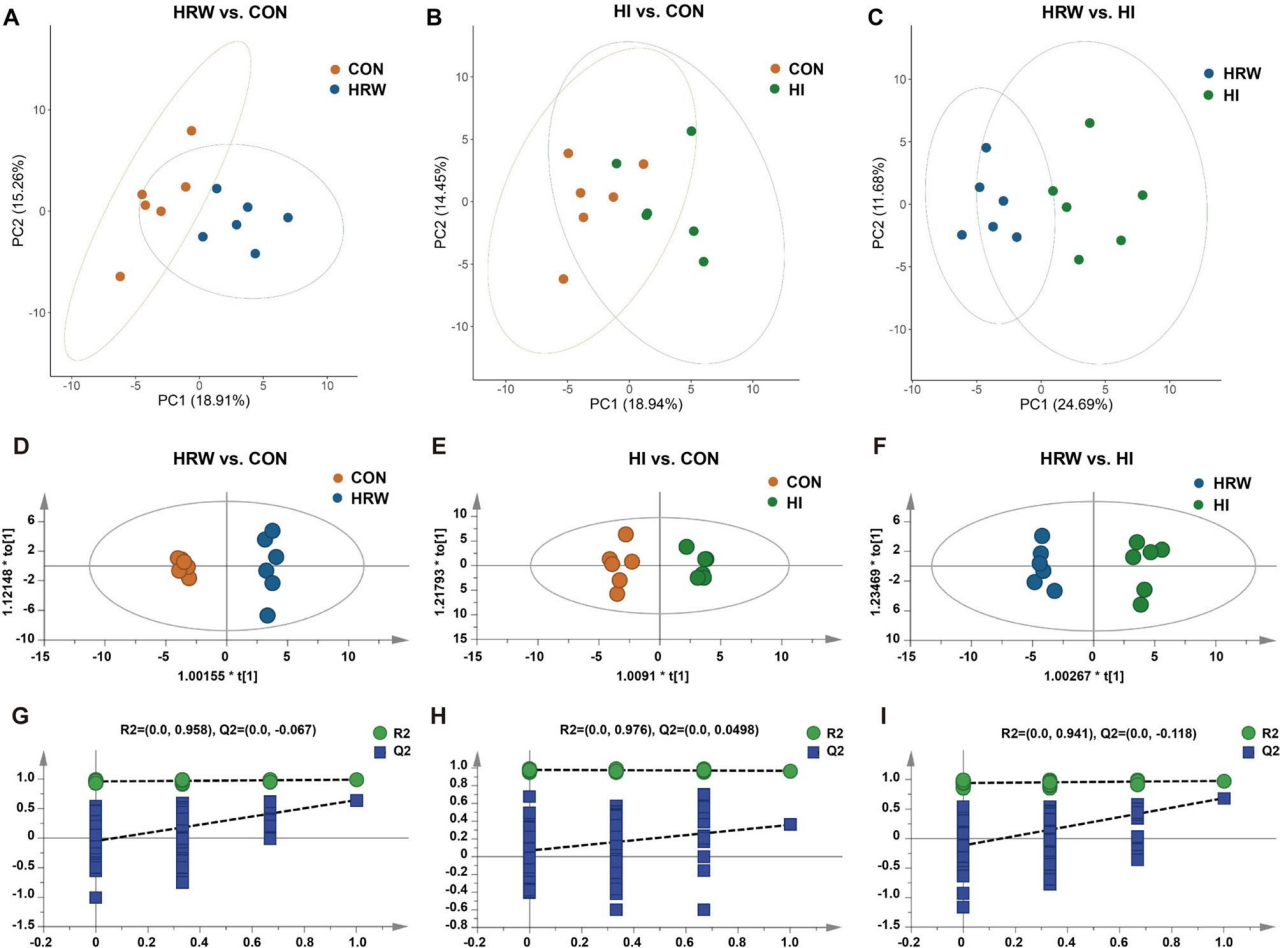
The difference on plasma metabolite profile between groups was identified by both PCA and OPLS-DA analysis. (**A**) PCA plot of HRW vs. CON; (**B**) PCA plot of HI vs. CON; (**C**) PCA plot of HRW vs. HI; (**D**) OPLS-DA score plot of HRW vs. CON; (**E**) OPLS-DA score plot of HI vs. CON; (**F**) OPLS-DA score plot of HRW vs. HI; (**G**–**I**) Validation plot obtained from 200 permutation tests, respectively.

To determine the effects of hydrogen intervention on plasma metabolites, LC–MS-based pseudotargeted metabolomics analysis was performed on fasting plasma samples. Eighty-six plasma metabolites were identified consisting of amino acids and their derivatives, intermediates in glycolysis and the citric acid cycle, lipid metabolites, nucleotide metabolites, urea cycle metabolites, carbohydrates, co-factors/vitamins, and hormones. To identified the difference in the levels of plasma metabolites between groups, Principal component analysis (PCA) and orthogonal partial least-squares discriminant analysis (OPLS-DA) were performed. PCA analysis showed that HRW and CON groups were clearly segregated by principle components (PC)1 (18.91%) and 2 (15.26%) (Fig. 2A). The segregation of HI and CON (PC1: 18.94%, PC2: 14.45%, Fig. 2B), as well as HRW and HI (PC1: 24.69%, PC2: 11.68%, Fig. 2C) was also observed. The OPLS-DA model from the plasma metabolic profile showed a good discrimination between HRW and CON (R2X = 0.262, R2Y = 0.991, Q2 = 0.645, Fig. 2D,G), HI and CON (R2X = 0.269, R2Y = 0.968, Q2 = 0.359, Fig. 2E,H), HRW and HI (R2X = 0.325, R2Y = 0.976, Q2 = 0.684, Fig. 2F,I).

**Figure 3:**
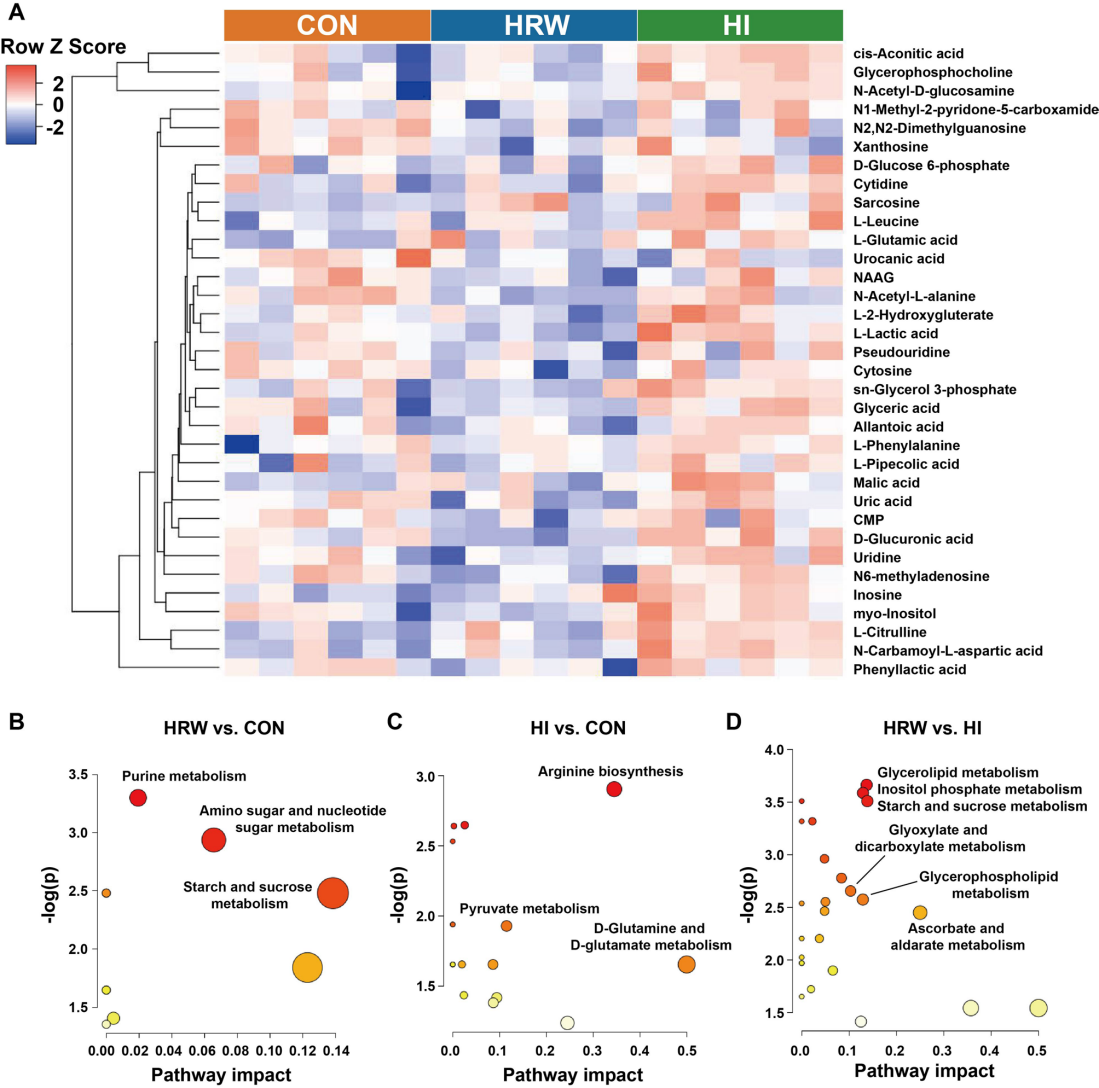
Analysis of differential metabolites in plasma samples among three groups. (**A**) The hierarchical clustering results for differential metabolites. (**B**–**D**) The functional enrichment analysis for differential metabolites between two groups. *NAAG* *N*-Acetylaspartylglutamic acid, *CMP* Cytidine 5′-monophosphate.

Differential metabolites (DMs) were selected by using the cutoff of OPLS-DA VIP score > 1.0 with a p value < 0.05 in the fold change of expression level between any two of the three groups. Thirty-five DMs were identified as shown in Supplementary Table 1. Compared with the control group, there are 14 and 10 DMs in HRW and HI group respectively. Twenty-two DMs were identified between HRW and HI group. Compared the control group, all the DMs were down-regulated in HRW group, while all the DMs were up-regulated in HI group. Compared HRW group, all the DMs were up-regulated in HI group. As shown in Fig. 3A, the dendrogram of hierarchical clustering showed the plasma samples in HRW group was clustered separately from the control group or HI group, however, the difference between HI group and the control group was much smaller. The pathway enrichment analysis based on metabolite quantitative alterations was performed by the MetaboAnalyst 5.0 (http://www.metaboanalyst.ca). The metabolic pathways with impact value > 0.1 and − log(p) > 2.0 are considered the most relevant pathways involved in the conditions under study. The results showed that the DMs between HRW and control group were mainly concentrated in starch and sucrose metabolism (Fig. 3B), the DMs between HI and control group were mainly involved in arginine biosynthesis (Fig. 3C), the DMs between HRW and HI group were mainly enriched in glycerolipid metabolism, inositol phosphate metabolism, starch and sucrose metabolism, glyoxylate and dicarboxylate metabolism, and ascorbate and aldarate metabolism (Fig. 3D).

### Effects of hydrogen intervention on faecal microbiota profiles of rats

**Figure 4:**
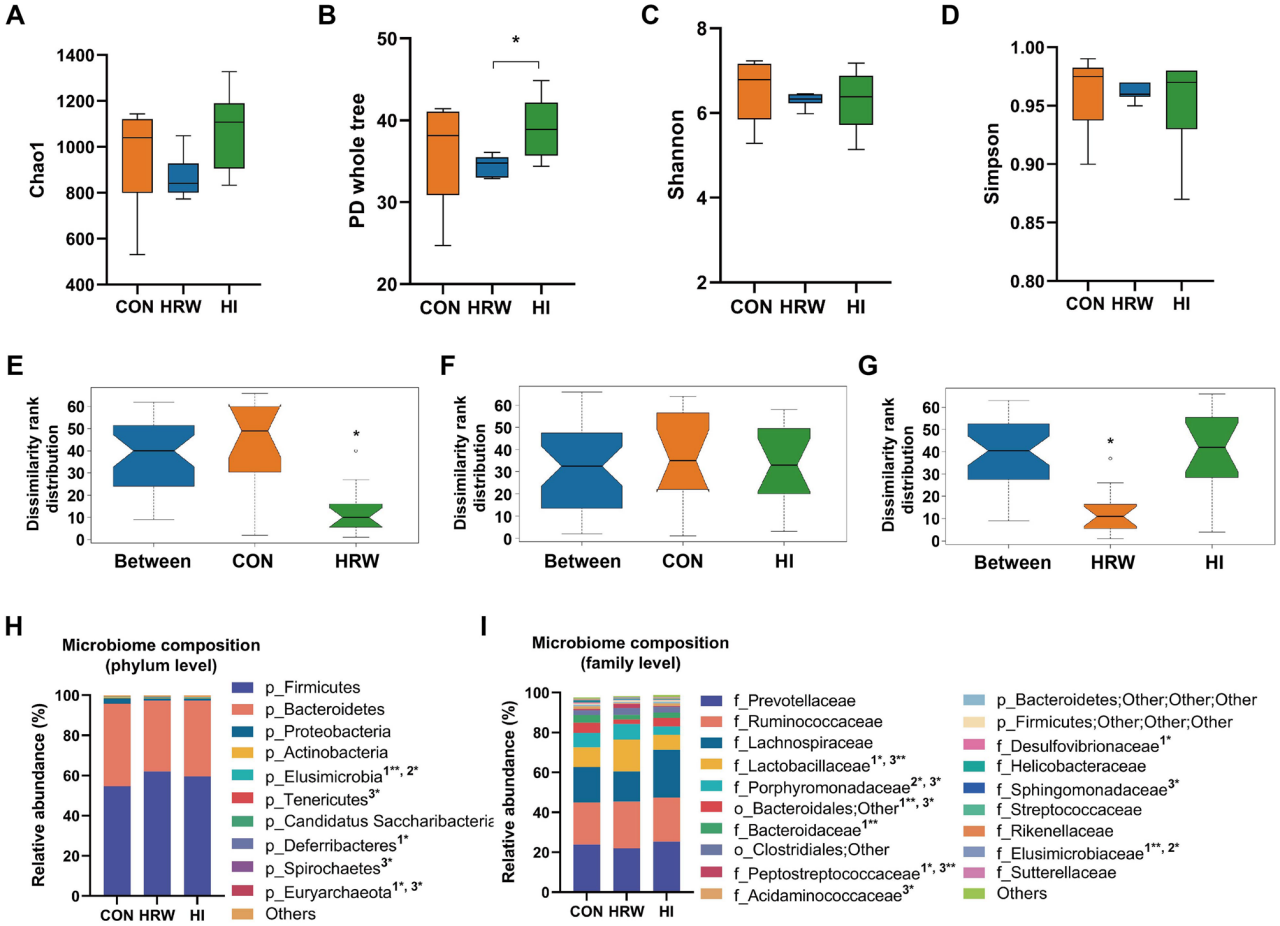
Analysis of bacterial community composition among three groups. (**A**–**D**) Alpha-diversity of bacterial community was measured by Chao1 (**A**), PD whole tree (**B**), Shannon (**C**), and Simpson (**D**); (**E**–**G**) ANOSIM was performed to quantitatively compare the bacterial community differences between different groups. (**H**,**I**) Community bar-plot analysis shows relative abundance of microbiota in each group at the phylum level (**H**) and family level (**I**), n = 6, in each group.

To investigate the effects of hydrogen intervention on faecal microbiota structure, we analyzed the bacterial communities on the samples by targeted 16S rRNA gene (V3–V4 region) sequencing using the Illumina MiSeq. The high quality sequence count (CON: 35,896 ± 766, HRW: 36,063 ± 884, HI: 35,630 ± 845) and operational taxonomic units (OTUs) count (CON: 551 ± 76, HRW: 516 ± 11, HI: 559 ± 84) were similar in the three groups. Four measures of α-diversity [Chao1 (Fig. 4A), PD whole tree (Fig. 4B), Shannon (Fig. 4C), and Simpson (Fig. 4D)] all fail to report significant difference between the control group and HRW or HI group. However, the PD whole tree index was significantly higher in HI group than that in HRW group (Fig. 4B). Analysis of similarities (ANOSIM) was performed to quantitatively compare the bacterial community differences between different groups. As shown in Fig. 4E, ANOSIM revealed significant difference in the structure of gut microbiota between HRW and the control group (ANOSIM, r = 0.304, p = 0.005). Significant bacterial community differences was also observed between HRW and HI group (Fig. 4G, ANOSIM, r = 0.369, p = 0.003). However, no significant difference was observed between HI and the control group (Fig. 4F, ANOSIM, r = − 0.065, p = 0.65). The Wilcoxon rank-sum test was performed to identify differences in relative abundances of bacteria between the two groups. At the phyla levels, as shown in Fig. 4H, the dominant phyla of the three groups were *Firmicutes*, *Bacteroidetes*, *Proteobacteria*, and *Actinobacteria*. Compared with the controls, the relative percentage of *Elusimicrobia* was significantly increased in HRW group, while the proportion of *Deferribacteres* and *Euryarchaeota* were markedly decreased in HRW group. HI only induced markedly increase in the proportion of *Elusimicrobia*. Compared with HRW group, the proportion of *Elusimicrobia* was significantly lower and *Spirochaetes* and *Euryarchaeota* were markedly higher in HI group. At the family level, as shown in Fig. 4I, the dominant family of the faecal microbiota were *Prevotellaceae*, *Ruminococcaceae*, *Lachnospiraceae*, and *Lactobacillaceae*. The abundances of *Lactobacillaceae*, *Peptostreptococcaceae*, and *Elusimicrobiaceae* were increased, and the abundances of *Bacteroidaceae* and *Desulfovibrionaceae* were decreased in HRW group compared with the controls. The decreased abundance in *Porphyromonadaceae* and increased abundance in *Elusimicrobiaceae* were observed in HI group compared with the controls. Compared with HRW group, the increased abundance in *Acidaminococcaceae* and decreased abundances in some other phyla (e.g., *Lactobacillaceae*, *Porphyromonadaceae*, *Peptostreptococcaceae*, and *Sphingomonadaceae*) were observed in HI group. At the genus levels, as shown in Supplementary Table 2, compared with the control group, eleven and seven genera exhibited significantly different abundances in HRW and HI group respectively. Compared with HRW group, twenty-three genera showed significant differences in relative abundances of bacteria in HI group.

**Figure 5:**
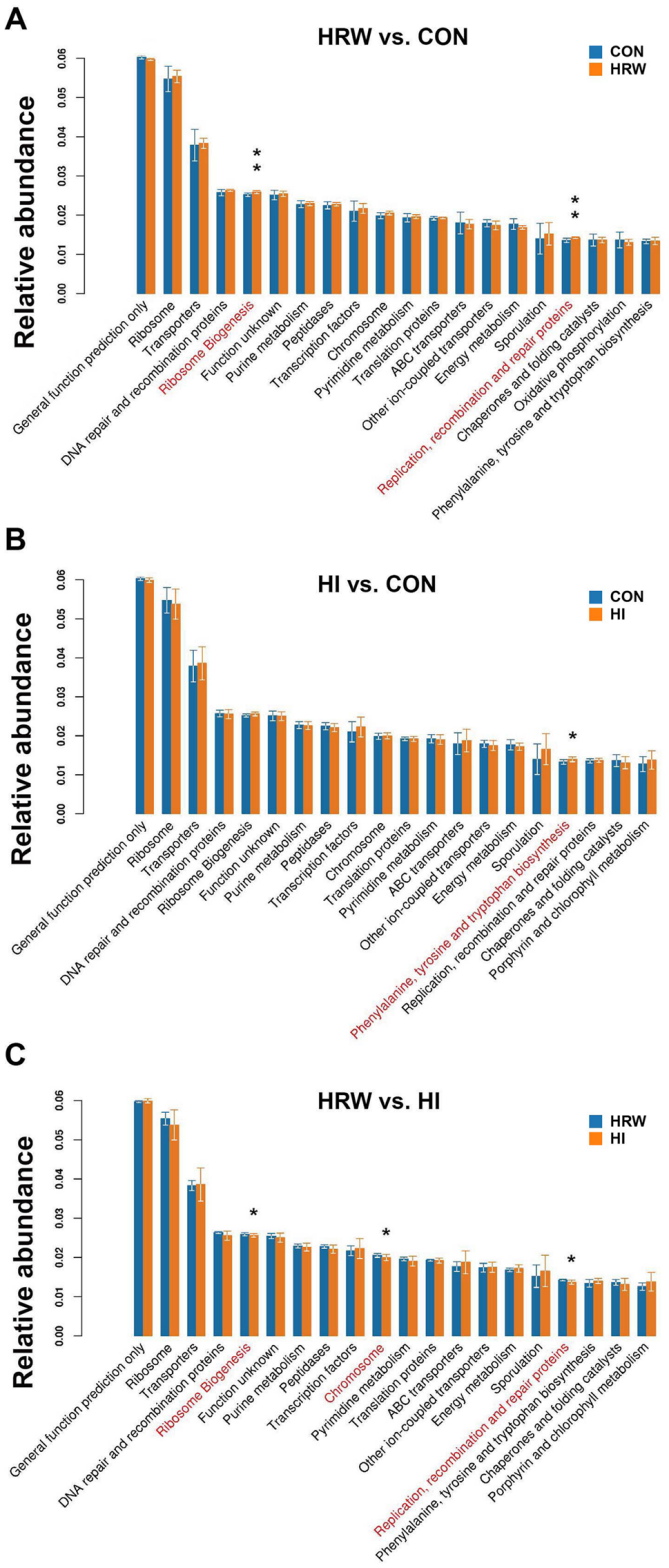
Differentially enriched KEGG pathways. The functional prediction at level-3 of KEGG pathway was performed by PICRUSt between HRW and Control group (**A**), HI and Control group (**B**), HRW and HI group (**C**).

To determine whether the changes in gut microbial taxa would alter the gut microbiota function, the functional prediction at level-3 of KEGG pathway was performed by PICRUSt. As shown in Fig. 5, compared with the control group, the faecal microbiota of rats from HRW group had elevated pathways involved in ribosome biogenesis and replication, recombination and repair proteins. HI group had significantly enriched pathway involved in phenylalanine, tyrosine and tryptophan biosynthesis. Compared with HRW group, HI group had reduced pathways involved in ribosome biogenesis, chromosome, and recombination and repair proteins.

### Correlations between the faecal microbiota and plasma metabolites

**Figure 6:**
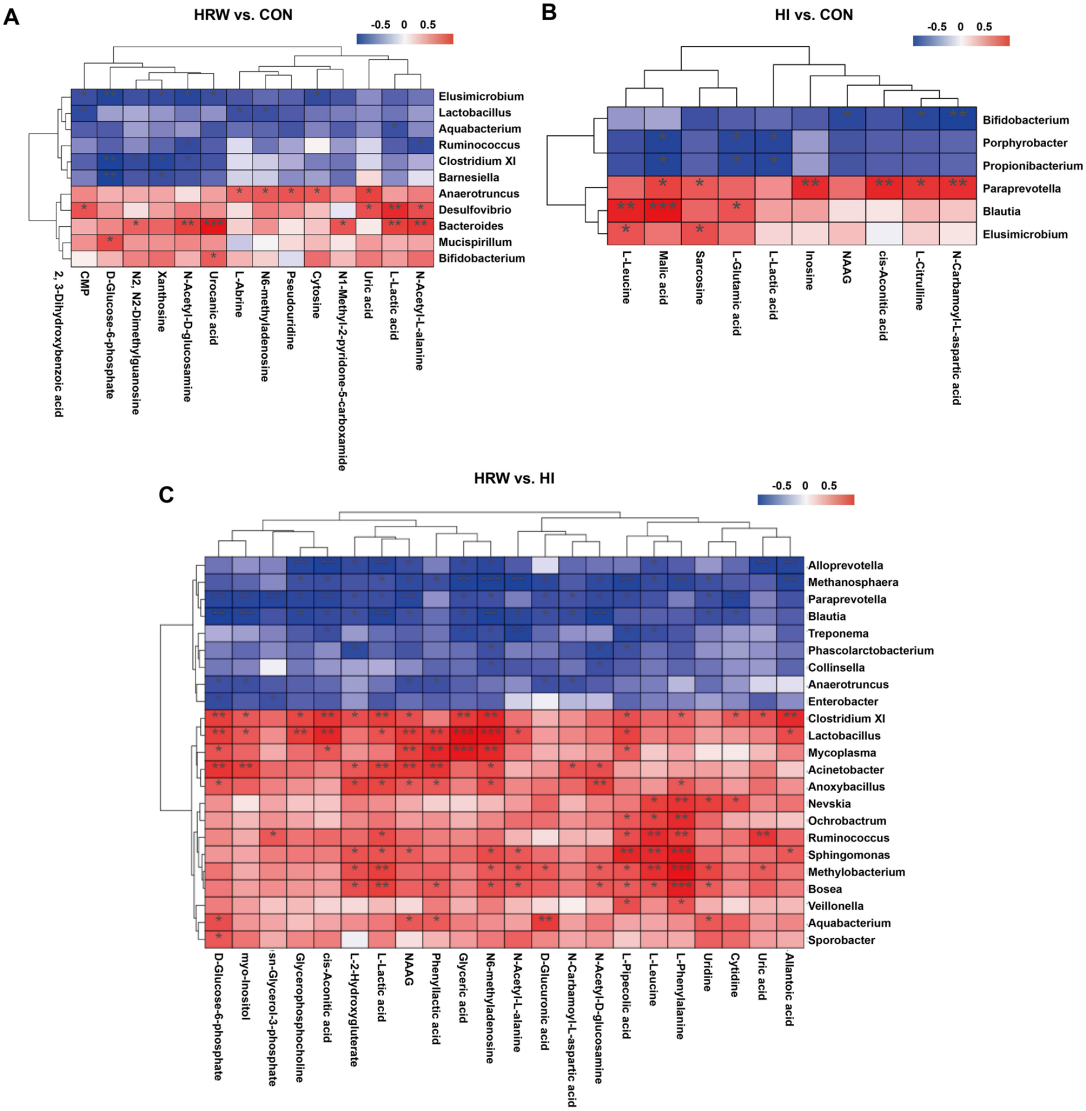
Correlation analyses between significantly different genera and differential plasma metabolites. Spearman’s correlation test was performed between HRW and Control group (**A**), HI and Control group (**B**), HRW and HI group (**C**).

To examine the possible relationship between differentially expressed plasma metabolites and altered gut microbiota at the genus level, multiomic analysis was performed by using Spearman’s correlation test. The results of Spearman’s correlation analysis between HRW and the control group are shown in Fig. 6A, the abundance of urocanic acid had the strongest correlation with *Bacteroides* (r = 0.944, p < 0.0001). l-lactic acid showed comparable moderate positive correlations with *Desulfovibrio* and *Bacteroides*. *N*-Acetyl-l-alanine and *N*-Acetyl-d-glucosamine also showed moderate positive correlation with *Bacteroides* and *Elusimicrobium*. d-Glucose 6-phosphate showed moderate negative correlations with *Elusimicrobium*, *Clostridium XI*, and *Barnesiella*. Figure 6B showed the Spearman’s correlation analysis between HI and the control group, malic acid had the strongest correlation with *Blautia* (r = 0.879, p = 0.0002). l-leucine showed moderate positive correlation with *Blautia*. *N*-Carbamoyl-l-aspartic acid, cis-Aconitic acid, and inosine also showed moderate positive correlations with *Paraprevotella*. *N*-Carbamoyl-l-aspartic acid showed moderate negative correlation with *Bifidobacterium*. Figure 6C showed the Spearman’s correlation analysis between HRW and HI group, l-phenylalanine showed high positive correlations with *Sphingomonas* (r = 0.866, p = 0.0003), *Methylocacterium* (r = 0.921, p < 0.0001), and *Bosea* (r = 0.848, p = 0.0005). Glyceric acid also showed high positive correlations with *Lactobacillus* (r = 0.937, p < 0.0001) and *Mycoplasma* (r = 0.881, p = 0.0002). N6-methyladenosine showed high positive correlation with *Lactobacillus* (r = 0.895, p < 0.0001), while had strong negative correlation with *Methanosphaera* (r = − 0.834, p = 0.0007).

## Discussion

Our previous study reported that HI can induce significant decrease in BW of rats, while HRW did not^21^. Consistent with previous study, the results of this study also showed markedly decrease in BW in HI group, while no obvious effect was observed in HRW group. The metabolic cage experiments showed that HI could significantly decrease the food intake, water intake, defecation and urination. The decreased metabolic function may contributed to the reduction of BW in HI group. Our previous study also showed that both HRW intake and HI can induce significant changes in several serum biochemical parameters in normal rats^21^. To obtain a more comprehensive understanding of the metabolic alterations in response to HRW intake or HI, LC–MS based pseudotargeted metabolomics analysis was performed. The OPLS-DA models indicated clear separations between any two of the three groups based on their metabolomic responses. Compared with the control group, 14 and 10 DMs were identified in HRW and HI group, respectively. It is worth noting that all the DMs in HRW group were down-regulated, while all the DMs were up-regulated in HI group, 22 DMs were identified between HRW and HI groups, indicating that the modulatory effects of HRW intake on metabolism differ markedly from HI. Further functional enrichment analysis suggested the DMs were mainly involved in starch and sucrose metabolism and arginine biosynthesis in HRW and HI group, respectively.

Previous study showed that 4 weeks of HRW intake could significantly decrease the levels of blood glucose, lactate, and blood urea nitrogen (BUN) and exert antifatigue effects in chronic forced swimming mice^22^. It has also been reported that 3 months of HRW intake could markedly decrease the blood uric acid levels in male patients with hyperuricemia^23^. Consistent with these findings, our results also showed that 6 months of HRW intake could down-regulate plasma levels of d-glucose 6-phosphate, l-lactic acid, and uric acid. In addition, the plasma levels of nucleotides and their derivatives were also reduced after HRW intake, indicating the regulatory effects of HRW intake on nucleotides metabolism. Notably, all the changed nucleotides derivatives, including m^6^A, pseudouridine, and N2,N2-dimethylguanosine, were belong to RNA modifications. Among them, m^6^A is the most widespread epigenetic modification on mammalian mRNA and has been shown to act as a key regulator of numerous important biological processes in normal physiology and in disease, including cancer, heart failure, viral infection, and type 2 diabetes^24,25^. Pseudouridine was reported to undergo dynamic changes in response to serum starvation, hydrogen peroxide and heat shock in mammalian cells^24^. It has previously been reported that H_2_ administration could regulate expression of diverse genes^26^, our results suggest that molecular hydrogen may regulate gene expression by affecting epigenetic modifications. In addition, the plasma levels of *N*1-methyl-2-pyridone-5-carboxamide was significantly decreased by HRW intake. Nicotinamide adenine dinucleotide (NAD^+^) is an important coenzyme for redox reactions, making it central to energy metabolism^27^. Nicotinamide mononucleotide (NMN) is one of the substrates for NAD^+^ synthesis, which can be further metabolized to *N*1-methyl-2-pyridone-5-carboxamide. Thus, HRW intake may regulate energy metabolism via affecting NAD^+^ synthesis.

Unlike HRW intake, HI had an up-regulatory effects on plasma DMs. Six of those DMs, including l-citrulline, l-leucine, sarcosine, l-glutamic acid, *N*-carbamoyl-l-aspartic acid, and NAAG, were belong to amino acids and their derivatives. Among them, the plasma levels of l-citrulline showed the most considerable increase in HI group compared to the controls. It has been showed in rats that only the intestine produced circulating citrulline, and the increased citrulline levels may be caused by either increased production or decreased utilization^28^. The decreased utilization could arise from a decrease in clearance, i.e. renal failure. However, no significant change of creatinine plasma levels was observed in our study, indicating that the increased citrulline levels was not caused by the impairment of renal function. Previous study showed that the increase in plasma citrulline was correlated with protein absorption improvement in patients with short bowel syndrome (SBS) followed in the first year after resection^29^. We supposed that the increased citrulline plasma levels may be associated with the improvement of enterocyte function, although this need to be further investigated. HI also induced a significant increase in NAAG plasma levels. NAAG is the most prevalent and widely distributed dipeptide in the mammalian nervous system^30^. The levels of NAAG in plasma and cerebrospinal fluid (CSF) were much lower than those in brain tissues^31^. It is well established that the increase in NAAG is neuroprotective against *N*-methyl-d-aspartate (NMDA) receptor-mediated neurotoxicity, including ischemic brain injury^30^. HI was first reported to exert neuroprotective effects on ischemic stroke, and further studies also found its protective effects on other neurological impairment, including traumatic brain injury, subarachnoid hemorrhage, and neurodegenerative diseases^32^. Consistent with our results, the HI induced increase in NAAG was also observed in cortex tissues of mice with ischemic stroke^33^. Although the ability of molecular hydrogen to scavenge hydroxyl radicals may partly explain its neuroprotective effects, the regulatory effects on NAAG may also responsible for its protective benefits. In addition, the significant increase in plasma levels of two citric acid cycle intermediates, cis-aconitic acid and malic acid, were also observed in our study, indicating that HI may accelerate mitochondrial energy metabolism. *N*-carbamoyl-l-aspartic acid and inosine are intermediates of pyrimidine and purine metabolism, respectively. The increase in the two metabolites suggest that HI may have modulatory effects on nucleoside metabolism. The plasma levels of other DMs, including l-leucine, sarcosine, l-glutamic acid, and l-lactic acid, varied slightly by HI.

Recent studies have provided evidence that modulation of host gut microbiota may be one of the mechanisms contributing to the biological effects of exogenous hydrogen consumption. Qiu et al. showed that saturated hydrogen saline treatment could modulate the abundance of *Bacteroides*, *Bifidobacteria*, and *Lactobacillus* in feces, which may responsible for the improvement of lipid metabolism disorders in high-fat diet mice^34^. Jin et al. reported that sustained H_2_ release in the gut by hydrogen nanocapsule could increase the abundance of *Akkermansia muciniphila* and attenuate metabolic dysfunction-associated fatty liver disease^35^. In this study, HRW intake induced significant changes in the structure of gut microbiota, while no marked bacterial community differences was observed in HI group. Previous study showed that the peak of the hydrogen concentration in small intestine after oral intake of 5 ppm of HRW was approximately 20 times higher than that after inhalation of 4% hydrogen gas^36^. The significant difference in hydrogen concentration in intestine between HRW intake and HI may contribute to the different effects on microbiota composition.

In our study, HRW intake induced significant increase in the proportion of *Lactobacillus*, *Ruminococcus*, *Clostridium XI*, *Elusimicrobium*, *Barnesiella*, and *Aquabacterium*, and decrease in *Bacteroides*, *Anaerotruncus*, *Desulfovibrio*, *Mucispirillum*, and *Bifidobacterium*. *Lactobacillus* and *Bifidobacterium* are the most common probiotic bacteria with the reported beneficial effects including aid digestion, reduce constipation, resist infections, prevent traveler’s diarrhea and ameliorate intestinal bowel disease (IBD)^37^. In our study, HRW intake induced a significant increase in the abundance of *Lactobacillus*. The increased abundance of *Lactobacillus* induced by HRW intake may contribute to the beneficial effects of HRW. Although HRW also induced a marked decrease in *Bifidobacterium*, the relative abundances of *Bifidobacterium* is very low. A recent clinical study showed that drinking hydrogen-dissolved alkaline electrolyzed water (AEW) for two weeks induced an increase in *Bifidobacterium* in healthy volunteers^38^. The different pH values (HRW 7.5 vs. AEW 9.5) or duration of hydrogen treatment (HRW 6 months vs. AEW 2 weeks) may contribute to the different effects on the levels of *Bifidobacterium.* It has been shown that supplementation with *Ruminococcus flavefaciens* could attenuate the antidepressant effects of duloxetine on depressive-like behavior^39^, although the increased *Ruminococcus* has also been shown to be beneficial regarding antidepressant-induced constipation^39^. Previous study showed that 4 weeks of HRW intake could exert beneficial effects on depressive-like behavior in mice via suppression of the inflammasome activation^40^, the antidepressive effects of HRW may be diminished by long-term HRW intake induced increase in *Ruminococcus* according to our study. *Clostridium XI* belongs to class *Clostridia*, which have been reported to attenuate inflammation and allergic diseases^41^. It has also been demonstrated that *Clostridium* species can utilize indigestible polysaccharide and produce lots of short-chain fatty acids (SCFAs), which are now considered as key players in the interactions with the host that impact on health and disease, especially given recent evidence for their capacity to modify the epigenome and effects on tissues and organs beyond the gut^42^. The increase in *Clostridium XI* derived SCFAs may also contributed to the effects of HRW. In a study of 345 Chinese individuals, members of the genera *Bacteroides* has been shown to be more abundant in type II diabetic subjects compared to controls with normal glucose metabolism^43^. The improved glucose tolerance and hyperglycemia lowering effect of HRW intake have been previously reported^9,44^, which may be attributed by the HRW-induced decrease in *Bacteroides* levels. The results of the Spearman correlation analyses revealed a great number of significant correlations between the abundant of *Bacteroides* and DMs, including urocanic acid, l-lactic acid, *N*-acetyl-d-glucosamine, and *N*-Acetyl-l-tyrosine, however, the causal relationships between alterations in *Bacteroides* abundance and plasma DMs need to be further investigated. Although the levels of other genera, including *Elusimicrobium*, *Barnesiella*, *Aquabacterium*, *Anaerotruncus*, *Desulfovibrio*, *Mucispirillum*, and *Bifidobacterium* changed significantly, their relative abundance was very low.

Compared with HRW group, the changes in fecal microbiota were found to be much less in HI group. Among these changed genera, the abundances of *Blautia* and *Paraprevotella* were significantly increased. *Blautia* has been considered as a probiotic bacterium that occur widely in mammalian feces and intestines^45^. The increased abundances of *Blautia* may contributed to the effects of HI. The *Paraprevotella* genus has been found to be negatively correlated with the BMI index^46^. Consistently, the negative correlation was also observed between the abundance of *Paraprevotella* genus and the BW of rats. Spearman correlation analyses revealed a great number of significant correlations between the abundant of *Blautia* and DMs, Correlation analyses revealed significant negative correlations between the abundance of *Blautia* and DMs, including l-leucine and malic acid. The abundance of *Paraprevotella* was negatively correlated with DMs, including inosine, cis-aconitic acid, and *N*-carbamoyl-l-aspartic acid. The other changed genera, including *Elusimicrobium*, *Propionibacterium*, *Porphyrobacter*, *Methanosphaera*, and *Bifidobacterium,* all had relatively low abundance.

Although both HRW and HI have beneficial effects in various diseases, the underlying mechanisms of them may be different. For HRW intake, it has been reported that many kinds of diseases, especially gastrointestinal symptoms, such as constipation and diarrhea, could benefit from HRW. It is known that the gut microbiota plays an important role in health and disease, the modulatory effect of HRW on gut microbiota may significantly contribute to the improvement of these diseases. In our study, the results of metabolic cage experiments showed that HI induced significant changes in food/water intake of rats, which positively correlates with changes in defecation/urination, while no obvious effect was observed in HRW group. The reasons for the decline in food intake are multifactorial and involve both peripheral and central mechanisms^47^. Hyspler et al. used deuterium gas as a metabolic tracer to quantify hydrogen metabolism in the mammalian body and found that the deuterium can be oxidized to water^48^. In our study, the inhaled hydrogen gas may probably be oxidized to water, providing an endogenous supply of H_2_O, which might explain the decrease in the water intake of rats. Moreover, in our previous studies we have provided preliminary evidence that eukaryocytes have the ability of hydrogen metabolism, which may affect its metabolic activities^49,50^. It is, therefore, possible to hypothesize that HI may exert its biologic role through modulation of metabolic function of mitochondria, although this needs to be further investigated.

One limitation of the present study is that research on the effects of hydrogen intervention on faecal microbiota profiles was focused on the genus level, and did not conduct in-depth studies at the species or even strain levels. Another limitation is the lack of proven causal relationships between alterations in microbiota profiles and plasma metabolites. In addition, further study should also evaluate the effects of hydrogen intervention on the production of SFCAs, which play a key role in microbiota-host interactions.

Collectively, the results of this study could provide basic data for further research on hydrogen medicine. Our results also shed light on the effects of different routes of hydrogen intervention on microbiota profiles, which may significantly contributed to the therapeutic effects of hydrogen in various diseases.

## Materials and methods

### Animals and experimental design

The methods of animal experiment has been reported in our previous study as follows^21^: eighteen 3-week-old male Sprague–Dawley rats weighing 40–50 g were purchased from the Vital River Laboratory Animal Technology Co., Ltd (Beijing, China). Rats were housed under a constant temperature at 22–25 °C with a 12 h light–dark cycle and maintained on a normal diet. All procedures were approved by the Institutional Animal Experiment Committee of the Chinese PLA General Hospital and were conducted in compliance with the Regulations for the Administration of Affairs Concerning Experimental Animals (China) and the ARRIVE guidelines. Before the experiment, rats were adapted to laboratory conditions for one week. Rats were then randomly divided into three groups (6 in each group): (1) Control group: rats were maintained under normal conditions; (2) HRW group: rats were given HRW by oral intake for 1 h each time, two times per day; (3) HI group: rats were treated with HI (4%) for 1 h each time, two times per day. The experiment was last for six months. Fresh feces samples were collected via sterile operation before sacrifice, and were immediately stored at − 80 °C for microbiota analysis. After euthanasia, blood samples were immediately harvested from the portal vein. Plasma samples were obtained by centrifuging the whole blood at 1500*g* for 10 min at 4 °C, and stored at − 80 °C for pseudotargeted metabonomic analysis.

### Hydrogen rich water preparation

HRW (H_2_ concentration > 800 µM) was kindly provided by Shenzhen Kelieng Biomedical Co. Ltd. (Shenzhen, China) and stored under atmospheric pressure at 23 ± 2 °C in a stainless steel bucket (KLE-8). The hydrogen concentration was monitored using a hydrogen electrode (Unisense A/S, Aarhus, Denmark), ensuring that the hydrogen concentration of HRW for rats was maintained above 800 µM.

### Inhalation of hydrogen gas

Rats were placed in a transparent closed box (72 × 53 × 45 cm, length × width × height) connected to the hydrogen gas generator which composed of an Oxy-Hydrogen Machine (SG-3000; Gang’an Health Management [Beijing] Co., Ltd., Beijing, China) and a gas mixer, allowed to spontaneous respiration (4% H_2_, 96% air containing 21% O_2_) for 1 h each time, and two times per day. The concentration of hydrogen and oxygen in the closed box was monitored by Thermal trace GC ultra-gas chromatography (Thermo Fisher, MA, USA).

### Metabolic cage studies

Male Sprague–Dawley rats (220 ± 20 g) were purchased from the Vital River Laboratory Animal Technology Co., Ltd (Beijing, China) and randomly divided into three groups (n = 8 in each group): CON, HRW, and HI group. Metabolic function of rats were monitored during 4 weeks. Rats were housed individually in metabolic cages (Techniplast, UK) once a week. Food intake was calculated by the remaining weight of the food from the weight of food provided on the previous day. Water intake was calculated by subtracting the amount of water left from the measured amount of water provided on the previous day. The feces and urine during the 24 h was collected and measured. The BW was monitored during the four weeks. The data were analysed by two-way ANOVA repeated measures with Tukey’s post hoc test using GraphPad Prism 8.0.2 and presented as mean ± SD. Spearman correlation analysis was performed using GraphPad Prism 8.0.2.

### LC–MS based pseudotargeted metabolomics analysis

To identify the DMs in plasma, the pseudo-targeted metabolomics approach was performed, which allows to identify and quantify 200 plasma metabolites. The plasma samples were thawed at 4 °C and thoroughly vortexed. For each sample, 100 µL was taken and transferred into a 1.5 mL Eppendorf tube. Then 400 µL ice-cold methanol/acetonitrile (1:1, v/v) was added. The samples were mixed by vigorous vortexed, sonicated in an ice-cold water bath sonicator for 20 min, and kept at − 20 °C for 1 h. Next, the samples were centrifuged at 14,000*g* for 20 min at 4 °C. The supernatant was transferred into a glass sampling vial and vacuum-dried at 4 °C. Then 100 µL of acetonitrile/water (1:1, v/v) was added before centrifugation at 14,000*g* for 20 min at 4 °C. The resultant supernatants were used for metabolomics analysis. The quality control (QC) samples were prepared by mixing equal volumes of all plasma samples with all other steps as described above.

The chromatographic separation of plasma was carried out on a Waters I-class liquid chromatography system with ACQUITY UPLC BEH Amide column (1.7 µm, 2.1 × 100 mm column, Waters). The mobile phase system consists of 25 mM ammonium acetate + 25 mM ammonia (pH 9.75) (A) and acetonitrile (B). Column temperature was maintained at 40 °C, and flow rate was 0.3 mL/min. Sample injection volume was 2 µL. The linear gradient for mobile phase B was as follows: 0–1 min 95% B; 1–14 min, 95% to 65%; 14–16 min, 65% to 40%; 16–18 min, 40% B; 18–18.1 min, 40% to 95%; 18.1–23 min, 95% B. The QC samples were injected at regular intervals (every 6 samples) throughout the analytical run.

Mass data acquisition was performed using a 5500 QqQ MS (AB SCIEX). Detection was performed through ESI positive and negative modes. The MS parameters were set as follows: sheath gas temperature, 350 °C; dry gas temperature, 350 °C; sheath gas flow, 11 L/min; dry gas flow, 10 L/min; capillary voltage, 4000 V or − 3500 V in positive or negative modes, respectively; nozzle voltage, 500 V; and nebulizer pressure, 30 psi. Multiple reaction monitoring (MRM) mode was used for detection. The dwell time of each ion pair was 3 ms, and the total cycle time was 1.263 s. The original MRM-based metabolomics data were analyzed using MRMAnalyzer (R) as previously reported^34^.

Unsupervised PCA analysis was first performed to visualize the general trends for all the samples. Then, a supervised OPLS-DA model was used to classify the samples and find the relevant variables related to the sample grouping. SIMCA-p software (version 14.1, https://www.sartorius.com) was used to perform PCA and OPLS-DA analysis. The model was validated with 200 random permutations to assess the predictive variation of the model. Variable importance in projections (VIP) scores obtained from the OPLS-DA model were used to assess the contribution of each variable to the established model. Metabolites that had VIP > 1 and p < 0.05 are identified as significantly changed metabolites. MetaboAnalyst 5.0 (https://www.metaboanalyst.ca/) was used for the functional enrichment analysis of the disturbed metabolites. All p values were corrected using Benjamini–Hochberg multiple test correction.

### 16S rRNA gene sequencing and microbiota analysis

Total genomic DNA was isolated from feces using the QIAamp Fast DNA Stool Mini Kit (QIAGEN, Germany) following the manufacturer’s instructions. The quality and quantity of the extracted DNA were measured using a NanoDrop ND-2000 spectrophotometer (Thermo Fisher Scientific, USA). The V3-V4 region of the 16S rRNA gene were amplified with the forward primer 5′-ACTCCTACGGGAGGCAGCA-3′, and the reverse primer 5′-GGACTACHVGGGTWTCTAAT-3′. The sample-specific barcodes were added to the ends of the primers. The PCR program was set as follows: 98 °C 10 min, 25 cycles of 98 °C 15 s, 55 °C 30 s, 72 °C 30 s, and 72 °C 5 min. The products of amplification were purified and then sequenced on Illumina MiSeq platform with MiSeq Reagent Kit v3.

The sequencing data were analyzed using QIME package (version 1.9.1). High-quality paried-end reads (quality score ≥ 20) were assembled into tags by using FLASH (version 1.2.11). Tags with > 97% sequences identity were clustered into OTUs using USEARCH (version 10.0), and UCHIME (version 8.1) was utilized to identify and remove chimeric sequences. A representative sequence of each OTU was selected and subjected to BLAST to assign taxonomic classification using SILVA database (version 132).

The alpha diversity indices were calculated by QIIME (version 1.9.1). A one-way analysis of similarity (ANOSIM) was performed to determine the differences in bacterial communities among groups. The differences in Alpha diversity indexes and phyla, family, and genera relative abundances between groups were calculated by use of the Independent-sample *t*-test (for the normally distributed data) or Wilcoxon rank-sum test (for the non-normally distributed data). A p value < 0.05 was considered statistically significant. Phylogenetic Investigation of Communities by Reconstruction of Unobserved States (PICRUSt) was used to obtain relative Kyoto Encyclopedia of Genes and Genomes (KEGG) pathway abundance information^51–54^.

### Spearman multi-omic correlation analysis

Spearman correlation between the relative abundance of genera and the level of plasma metabolites was calculated in R software (version 3.2.1, https://www.r-project.org/) and visualized using ComplexHeatmap package (version 2.9.3, http://bioconductor.org/packages/release/bioc/html/ComplexHeatmap.html) in R software (version 3.2.1).

## Supplementary Information

Supplementary Tables.

## Data Availability

The data that support the findings of this study are available from the corresponding author upon reasonable request.
