# Supplementary material for: Different effects of hydrogen-rich water intake and hydrogen gas inhalation on gut microbiome and plasma metabolites of rats in health status
Source: Sci Rep. 2022 May 4;12:7231. doi: 10.1038/s41598-022-11091-1 (PMC9068821; doi:10.1038/s41598-022-11091-1)
Supplement: Supplementary file 1 — Supplementary Tables. [file 41598_2022_11091_MOESM1_ESM.docx]

Supplementary Table 1 Differentially expressed plasma metabolites between two groups

|  | Compound | Molecular formula | Rt (min) | VIP | Fold change | p value |
| --- | --- | --- | --- | --- | --- | --- |
| HRW/Control | N-acetyl-D-glucosamine | C_8_H_15_NO_6_ | 7.54 | 2.05 | 0.75 | 0.002 |
|  | D-glucose 6-phosphate | C_6_H_13_O_9_P | 13.82 | 1.89 | 0.71 | 0.003 |
|  | N2,N2-dimethylguanosine | C_12_H_17_N_5_O_5_ | 5.31 | 1.85 | 0.63 | 0.008 |
|  | xanthosine | C_10_H_12_N_4_O_6_ | 8.64 | 1.71 | 0.64 | 0.010 |
|  | N6-methyladenosine (m^6^A) | C_11_H_15_N_5_O_4_ | 3.59 | 1.70 | 0.57 | 0.016 |
|  | urocanic acid | C_6_H_6_N_2_O_2_ | 8.45 | 1.69 | 0.83 | 0.020 |
|  | L-lactic acid | C_3_H_6_O_3_ | 5.91 | 1.68 | 0.86 | 0.023 |
|  | cytosine | C_4_H_5_N_3_O | 5.80 | 1.67 | 0.79 | 0.014 |
|  | N-Acetyl-L-tyrosine | C_11_H_13_NO_4_ | 5.89 | 1.65 | 0.79 | 0.021 |
|  | uric acid | C_5_H_4_N_4_O_3_ | 9.04 | 1.56 | 0.69 | 0.047 |
|  | CMP | C_9_H_14_N_3_O_8_P | 12.46 | 1.51 | 0.71 | 0.035 |
|  | pseudouridine | C_9_H_12_N_2_O_6_ | 6.92 | 1.51 | 0.82 | 0.032 |
|  | N1-methyl-2-pyridone-5-carboxamide | C_7_H_8_N_2_O_2_ | 2.21 | 1.43 | 0.62 | 0.034 |
|  | L-abrine | C_12_H_14_N_2_O_2_ | 7.05 | 1.21 | 0.65 | 0.042 |
| HI/Control | L-citrulline | C_6_H_13_N_3_O_3_ | 12.01 | 2.14 | 2.86 | 0.008 |
|  | L-leucine | C_6_H_13_NO_2_ | 8.10 | 2.08 | 1.20 | 0.010 |
|  | N-carbamoyl-L-aspartic acid | C_5_H_8_N_2_O_5_ | 11.99 | 2.04 | 1.94 | 0.005 |
|  | inosine | C_10_H_12_N_4_O_5_ | 6.13 | 2.03 | 1.89 | 0.002 |
|  | malic acid | C_4_H_6_O_5_ | 11.15 | 1.82 | 1.46 | 0.028 |
|  | sarcosine | C_3_H_7_NO_2_ | 10.41 | 1.70 | 1.24 | 0.045 |
|  | L-glutamic acid | C_5_H_9_NO_4_ | 11.35 | 1.67 | 1.16 | 0.026 |
|  | L-lactic acid | C_3_H_6_O_3_ | 5.91 | 1.62 | 1.20 | 0.049 |
|  | cis-aconitic acid | C_6_H_6_O_6_ | 12.10 | 1.39 | 2.15 | 0.026 |
|  | N-acetylaspartylglutamic acid (NAAG) | C_11_H_16_N_2_O_8_ | 12.15 | 1.21 | 2.21 | 0.019 |
| HRW/HI | D-glucose 6-phosphate | C_6_H_13_O_9_P | 13.82 | 1.85 | 0.55 | 0.001 |
|  | glyceric acid | C_3_H_6_O_4_ | 8.45 | 1.82 | 0.75 | 0.001 |
|  | L-lactic acid | C_3_H_6_O_3_ | 5.91 | 1.78 | 0.71 | 0.003 |
|  | N6-methyladenosine | C_11_H_15_N_5_O_4_ | 3.59 | 1.73 | 0.52 | 0.001 |
|  | N-acetylaspartylglutamic acid (NAAG) | C_11_H_16_N_2_O_8_ | 12.15 | 1.70 | 0.47 | 0.007 |
|  | glycerophosphocholine | C_8_H_20_NO_6_P | 12.08 | 1.68 | 0.21 | 0.049 |
|  | sn-glycerol 3-phosphate | C_3_H_9_O_6_P | 12.38 | 1.63 | 0.76 | 0.009 |
|  | cis-aconitic acid | C_6_H_6_O_6_ | 12.10 | 1.63 | 0.37 | 0.001 |
|  | myo-inositol | C_6_H_12_O_6_ | 12.06 | 1.57 | 0.62 | 0.016 |
|  | L-leucine | C_6_H_13_NO_2_ | 8.10 | 1.52 | 0.83 | 0.010 |
|  | N-carbamoyl-L-aspartic acid | C_5_H_8_N_2_O_5_ | 11.99 | 1.51 | 0.62 | 0.020 |
|  | L-phenylalanine | C_9_H_11_NO_2_ | 7.45 | 1.41 | 0.88 | 0.027 |
|  | L-pipecolic acid | C_6_H_11_NO_2_ | 9.10 | 1.40 | 0.78 | 0.022 |
|  | L-2-hydroxygluterate | C_5_H_8_O_5_ | 10.83 | 1.40 | 0.74 | 0.021 |
|  | N-acetyl-D-glucosamine | C_8_H_15_NO_6_ | 7.54 | 1.37 | 0.79 | 0.015 |
|  | phenyllactic acid | C_9_H_10_O_3_ | 1.27 | 1.36 | 0.45 | 0.044 |
|  | uridine | C_9_H_12_N_2_O_6_ | 4.35 | 1.35 | 0.58 | 0.013 |
|  | uric acid | C_5_H_4_N_4_O_3_ | 9.04 | 1.32 | 0.65 | 0.033 |
|  | cytidine | C_9_H_13_N_3_O_5_ | 6.94 | 1.32 | 0.80 | 0.013 |
|  | N-acetyl-L-alanine | C_5_H_9_NO_3_ | 6.90 | 1.26 | 0.80 | 0.039 |
|  | allantoic acid | C_4_H_8_N_4_O_4_ | 10.09 | 1.26 | 0.79 | 0.021 |
|  | D-glucuronic acid | C_6_H_10_O_7_ | 11.00 | 1.21 | 0.85 | 0.039 |

Supplementary Table 2 Significant differences in the relative abundances of bacteria at the genus level between two groups.

|  | Taxa | Relative abundance of Group 1 | Relative abundance of Group 2 | p value |
| --- | --- | --- | --- | --- |
| Group 1: HRW  Group 2： Control | *Lactobacillus* | 1.60×10^-1^±0.38×10^-1^ | 0.99×10^-1^±0.74×10^-1^ | 0.047 |
|  | *Ruminococcus* | 0.85×10^-1^±0.46×10^-1^ | 0.41×10^-1^±0.32×10^-1^ | 0.047 |
|  | *Bacteroides* | 0.22×10^-1^±0.05×10^-1^ | 0.39×10^-1^±0.12×10^-1^ | 0.008 |
|  | *Clostridium XI* | 2.00×10^-2^±0.99×10^-2^ | 0.67×10^-2^±0.53×10^-2^ | 0.013 |
|  | *Elusimicrobium* | 3.69×10^-3^±2.15×10^-3^ | 0.65×10^-3^±0.68×10^-3^ | 0.004 |
|  | *Anaerotruncus* | 0.29×10^-3^±0.51×10^-3^ | 0.43×10^-3^±0.24×10^-3^ | 0.032 |
|  | *Desulfovibrio* | 0.48×10^-4^±0.70×10^-4^ | 2.37×10^-4^±1.59×10^-4^ | 0.036 |
|  | *Barnesiella* | 6.48×10^-4^±5.49×10^-4^ | 2.08×10^-4^±2.42×10^-4^ | 0.047 |
|  | *Mucispirillum* | 0 | 1.20×10^-4^±1.68×10^-4^ | 0.037 |
|  | *Bifidobacterium* | 0 | 3.06×10^-5^±4.32×10^-5^ | 0.037 |
|  | *Aquabacterium* | 5.51×10^-5^±8.62×10^-5^ | 0 | 0.037 |
| Group 1: HI  Group 2： Control | *Blautia* | 0.77×10^-2^±1.32×10^-2^ | 0.20×10^-2^±0.47×10^-2^ | 0.022 |
|  | *Paraprevotella* | 1.24×10^-2^±1.09×10^-2^ | 0.33×10^-3^±0.42×10^-3^ | 0.001 |
|  | *Elusimicrobium* | 2.03×10^-3^±1.49×10^-3^ | 0.65×10^-3^±0.68×10^-3^ | 0.033 |
|  | *Propionibacterium* | 0 | 0.30×10^-3^±0.57×10^-3^ | 0.037 |
|  | *Porphyrobacter* | 0 | 0.25×10^-3^±0.43×10^-3^ | 0.037 |
|  | *Methanosphaera* | 0.93×10^-4^±1.40×10^-4^ | 0.37×10^-4^±0.91×10^-4^ | 0.044 |
|  | *Bifidobacterium* | 0 | 0.31×10^-4^±0.43×10^-4^ | 0.037 |
| Group 1: HRW  Group 2： HI | *Lactobacillus* | 1.60×10^-1^±0.38×10^-1^ | 0.75×10^-1^±0.22×10^-1^ | 0.001 |
|  | *Ruminococcus* | 0.85×10^-1^±0.46×10^-1^ | 0.50×10^-1^±0.51×10^-1^ | 0.032 |
|  | *Clostridium XI* | 2.00×10^-2^±0.99×10^-2^ | 0.38×10^-2^±0.29×10^-2^ | 0.001 |
|  | *Alloprevotella* | 1.24×10^-2^±0.84×10^-2^ | 4.75×10^-2^±3.43×10^-2^ | 0.021 |
|  | *Phascolarctobacterium* | 0.43×10^-2^±0.47×10^-2^ | 1.36×10^-2^±0.80×10^-2^ | 0.032 |
|  | *Sphingomonas* | 1.57×10^-3^±1.88×10^-3^ | 0.21×10^-3^±0.45×10^-3^ | 0.015 |
|  | *Veillonella* | 1.25×10^-3^±0.56×10^-3^ | 0.82×10^-3^±0.98×10^-3^ | 0.047 |
|  | *Mycoplasma* | 0.88×10^-3^±0.59×10^-3^ | 0.20×10^-3^±0.16×10^-3^ | 0.013 |
|  | *Methylobacterium* | 0.88×10^-3^±0.94×10^-3^ | 0.69×10^-4^±1.51×10^-4^ | 0.019 |
|  | *Bosea* | 5.24×10^-4^±4.33×10^-4^ | 0.69×10^-4^±1.51×10^-4^ | 0.028 |
|  | *Sporobacter* | 3.41×10^-4^±2.40×10^-4^ | 1.36×10^-4^±0.94×10^-4^ | 0.047 |
|  | *Ochrobactrum* | 3.29×10^-4^±3.47×10^-4^ | 0.38×10^-4^±0.92×10^-4^ | 0.045 |
|  | *Anaerotruncus* | 2.88×10^-4^±5.09×10^-4^ | 4.58×10^-4^±2.44×10^-4^ | 0.047 |
|  | *Nevskia* | 1.56×10^-4^±2.50×10^-4^ | 0 | 0.037 |
|  | *Paraprevotella* | 1.53×10^-4^±1.43×10^-4^ | 1.24×10^-2^±1.09×10^-2^ | 0.002 |
|  | *Anoxybacillus* | 1.47×10^-4^±1.30×10^-4^ | 0.19×10^-4^±0.46×10^-4^ | 0.030 |
|  | *Blautia* | 1.04×10^-4^±1.34×10^-4^ | 0.77×10^-2^±1.32×10^-2^ | 0.002 |
|  | *Acinetobacter* | 0.55×10^-4^±1.01×10^-4^ | 0 | 0.037 |
|  | *Aquabacterium* | 0.55×10^-4^±0.86×10^-4^ | 0 | 0.037 |
|  | *Treponema* | 0.43×10^-4^±0.59×10^-4^ | 1.77×10^-4^±1.38×10^-4^ | 0.036 |
|  | *Enterobacter* | 0 | 0.28×10^-3^±0.64×10^-3^ | 0.037 |
|  | *Methanosphaera* | 0 | 0.93×10^-4^±1.40×10^-4^ | 0.005 |
|  | *Collinsella* | 0 | 0.25×10^-4^±0.31×10^-4^ | 0.037 |
